# The Mortuary Unit

**Published:** 1913-01-11

**Authors:** 


### THE MAKING OF A MODERN HOSPITAL.

### The Mortuary Unit.

INSTITUTIONAL INTERESTS—II.

In planning a good mortuary unit certain special interests must be considered. These are: first, the interests of the institution, and, secondly, those of the public. Where a hospital has a teaching school attached to it the planning of the mortuary block is not quite so simple a matter as when the unit is intended to serve a smaller institution that has no teaching school attached to it.

The block must be built on a suitable site. Usually if one were to judge by the arrangement of the mortuary block in most hospitals, the architect considers that any out-of-the-way corner will do for the dead-house, so long as it is a corner that is more or less private. But privacy is not the only thing to be taken into account. Generally the mortuary block is arranged at the extreme end of the hospital site, sometimes near the economic block, sometimes near the pathological buildings, with which it may be intimately associated.

### The Mortuary and the Post-Mortem Room.

For the sake of simplicity it may be well to differentiate between the mortuary proper and the post-mortem room, which is an indispensable adjunct to the pathological block. The former is essentially fitted for the smaller non-teaching institution; the latter usually belongs to the teaching hospital or to the large general hospital, which possesses a proper pathological department. But in the latter case it is imperative that the institutional interests—in this case the interests of the prosectors and the pathologists—should not be made superior to those of the public. Whether there is a pathological department or not the mortuary itself, with its attached waiting rooms, chapel, and undertakers' room, must be complete in itself.

Whatever site is fixed upon for the unit, care must be taken that the mortuary block is not directly overlooked by the pavilions or balconies of the ward, and that the approach to it is not commanded by the hospital walks frequented by patients or their friends. In modern hospitals it is almost the rule to have subterranean approaches to the mortuary building, which connect the dead-house with all the departments and with the wards, so that a corpse can be wheeled into the post-mortem room without attracting attention. The ordinary ward lift is sometimes used for the conveyance of the post-mortem room trolley, and it is even now no rare sight to see the porters carrying a bier towards a lift which a few minutes later may be used to convey a new patient to the ward. Exigencies of space and economy may cause such a system to be tolerated, but it is certainly not an ideal system. A better way is to have an extra lift, serving a number of wards and intended solely for the conveyance of the dead, for dirty linen, or for supplies, the ordinary ward lift being reserved for patients. It is needless to say that the removal of the dead body from the ward should be made with the utmost quietness and with the least possible publicity. It is still a moot question whether a special room for the dying is desirable in a hospital ward; where such is not provided there are obvious difficulties in the way of carrying out the corpse of a patient without at the same time informing all the other patients in the ward that a death has occurred. By attention to the little points—by ensuring that the mortuary trolley works noiselessly, by avoiding striking palls and trappings, and by screening as much as possible the approach to the bed—much may be done to ensure that desirable privacy. Once the bier or trolley is down in the subway the journey to the post-mortem room can be accomplished with entire privacy. Where no subways are provided the course of the bier through the hospital grounds is often an unnecessarily exciting spectacle to watching patients—a spectacle which ought to be prevented wherever possible.

The site for the mortuary should be selected not merely because it is not overlooked directly by the other hospital buildings tenanted by patients, but also on account of considerations of light and air and isolation. For the wards it is necessary that the buildings should be so grouped that their long axes are directed north-east and south-west, so as to ensure that they get the maximum amount of sunlight. Direct sunlight, however, is not necessary, or even desirable, in the mortuary; here reflected light is more useful, especially in the pathological departments, since direct sunlight considerably interferes at times with the proper examination of specimens. The necessary isolation must be provided for by arranging the mortuary block in such a manner that it is independent of the usual hospital approaches and exits. Thus the block should have its own approach from the street or roadway to admit of access by friends or relatives or by the undertaker's van or hearse. The closer this entrance is to the roadway the better, though care should be taken that the block itself is isolated from the outside world. This is best done by a wall with an inner row of trees, as is the case at the Virchow Hospital at Berlin, where the mortuary block stands in an ideal position, both so far as the institution and the outside world are concerned.

### An Overlooked Necessity.

The block is in charge of a special staff which in small hospitals consists usually of a post-mortem room porter or mortuary attendant, but in larger institutions comprises several attendants. No matter how few or how many attendants there are, adequate provision should be made for their comfort, as well as for that of those who have to work in the mortuary—students, demonstrators, and undertakers' men. This is a matter which is often entirely overlooked, and it is worth while to go into some detail with regard to it. It is a well-known fact that those who have to work in the post-mortem room or conduct autopsies find it necessary to ensure perfect and scrupulous cleanliness of their person. Such work generally leaves characteristic traces behind it; it is difficult to rid the hands of the post-mortem room smell, and more difficult still to get rid of that odour from the body emanations and excreta. That at least is the opinion of experienced post-mortem room attendants who have worked for years in hospital mortuaries. But it is also a fact that where proper provision is made to enable the attendants to cleanse themselves immediately after their work these temporary unpleasant concomitants of post-mortem work are by no means so conspicuous as they are in the case of those who are unable to make use of such provision for adequate cleansing.

In the newest type of post-mortem block the attendants' bathroom and lavatory are regarded as indispensable adjuncts. A proper supply of hot water is arranged for, and a full-length bath with attached shower is provided, together with a dressing room. Where the mortuary is used by a prosector similar arrangements must be made in duplicate for his comfort; when necessary the undertakers' men are allowed to use the attendants' lavatory. Adequate provision must be made, too, for occasional washing and sterilising of the hands; this is secured by having a couple of simple wall bracket basins—the older operating theatre model serves excellently—with special basins for antiseptic solutions. The dressing room must be arranged well away from the post-mortem room itself, and must be simply furnished so that it may also serve as a private room for the prosector. The special overalls worn while doing post-mortem examinations are kept in this room in a cupboard; after use they are thrown into the basket in the lavatory, whence they are collected by the attendant for washing in the institutional laundry. From the institutional point of view it is sometimes desirable to keep pathological specimens, or even bodies, for some time before dealing with them in the ordinary way. This is especially the case where medico-legal questions are involved. In foreign hospitals, where the legal post-mortem is frequent, the law prescribes certain formalities which are not prescribed for English institutions, since with us a legal autopsy is rarely performed in a hospital mortuary, but more usually in a public one. Nevertheless, it is desirable that every mortuary should possess an up-to-date refrigerating chamber where bodies and specimens can be kept for some time if necessary. Where a pathological block is attached to the institution, and where, moreover, a teaching school is in existence, it may be desirable to incorporate with this department a pickling room and an injecting chamber. This is the arrangement devised by Vandervelde and De Page in their model mortuary, of which we shall publish a ground-plan sketch. In English teaching hospitals it is more usual to separate the purely anatomical from the pathological departments and to have the pickling and injecting rooms arranged as adjuncts to the dissecting room; in hospitals abroad, which are also teaching centres, the whole pathological block is devoted to what is called anatomico-pathological teaching. To this rule, however, there are many exceptions, notably at Berlin, where the anatomical department is housed in a separate building near the veterinary school and the mortuary block of the Charité is reserved for pathological work. Even in such cases, however, it is difficult to draw a sharp line of demarcation, for the classes in operative surgery on the cadaver and on animals are held in the pathological block.

That, indeed, seems the best arrangement since it conduces to economy of space and material, especially where the pathological department uses a large number of animals for experimental purposes. The arrangement of these special rooms are best seen from the plans which we shall give in a future article. All that need be said here is that this side of the mortuary block represents more especially the institutional side, and should therefore be kept distinct from the mortuary proper. The latter, as a unit, consists essentially of three main divisions. The first is the post-mortem room, with its room for freshly brought in corpses, its examining room or post-mortem room proper, its viewing room, its chapel or receiving room, its undertakers' or coffin room, its waiting room for the patients' friends, and finally the lavatories attached to these rooms. In some hospitals it is the custom to place the corpses of those who have died of infectious diseases in a special viewing room, the body being guarded by a plate-glass partition; the best example of this in London is to be found in the mortuary block of the London Fever Hospital in Islington. Such a precaution seems unnecessary, but is probably regarded as an additional safeguard by the public and therefore can be retained. Of course, it is quite another matter when the corpse of a patient who has died of an infectious disease has to be handled by the attendants, prosector, students, or undertakers' men; in that case a special room reserved for infectious cases is absolutely necessary, and it is usual to place this special room close to the ordinary post-mortem room, but with a separate entrance and exit into the corridor so as to isolate it as much as possible.

The ventilation of the mortuary buildings is a matter which needs careful study. Probably the most efficient system is that which ensures direct natural ventilation. The theoretical objections to direct ventilation, however good they may seem when a hospital ward is concerned, do not hold when applied to the mortuary: first, because no night work is done in the department as a rule, and consequently there is no liability to the "night stagnation" which is such a feature of the ordinary ward that is "naturally" ventilated; and, secondly, because the admission of dust into the mortuary is not such an evil as it would be in the ward. On the other hand, with open windows and efficient natural ventilation, there is a fair chance of removing the unpleasant odours that are so characteristic of the post-mortem room; with a system of forced ventilation these odours are never got rid of, however frequently the air may be changed. This is particularly the case when the rooms are heated by low-pressure hot-water pipes. The ideal arrangement for the post-mortem room itself is natural ventilation and heating by means of open fires, either gas or coal.

In practice, however, it is found more convenient to have hot-water radiators for heating, and although these never work quite satisfactorily, so far as the comfort of those who have to work in the rooms are concerned, they are more economical and need less attention than open fires. The main thing in the ventilation of the rooms is that there should be a constant supply of fresh air entering low down and emerging higher up, so as to carry off the odours that are developed in the room. It is difficult to find a properly ventilated post-mortem room, simply because, hitherto, architects have not devoted their best consideration to the question of accelerating the exit of foul air from the building.

